# The importance of Rydberg orbitals in dissociative ionization of small hydrocarbon molecules in intense laser fields

**DOI:** 10.1038/s41598-017-04638-0

**Published:** 2017-06-30

**Authors:** Bethany Jochim, R. Siemering, M. Zohrabi, O. Voznyuk, J. B. Mahowald, D. G. Schmitz, K. J. Betsch, Ben Berry, T. Severt, Nora G. Kling, T. G. Burwitz, K. D. Carnes, M. F. Kling, I. Ben-Itzhak, E. Wells, R. de Vivie-Riedle

**Affiliations:** 1 J.R. Macdonald Laboratory, Department of Physics, Kansas State University, Manhattan, KS 66506 USA; 2 Department für Chemie, Ludwig-Maximilians-Universität München, Butenandt-Strasse 11, D-81377 München, Germany; 3 Department of Physics, Augustana University, Sioux Falls, SD 57197 USA; 4 Department für Physik, Ludwig-Maximilians-Universität München, Am Coulombwall 1, D-85748 Garching, Germany

## Abstract

Much of our intuition about strong-field processes is built upon studies of diatomic molecules, which typically have electronic states that are relatively well separated in energy. In polyatomic molecules, however, the electronic states are closer together, leading to more complex interactions. A combined experimental and theoretical investigation of strong-field ionization followed by hydrogen elimination in the hydrocarbon series C_2_D_2_, C_2_D_4_ and C_2_D_6_ reveals that the photofragment angular distributions can only be understood when the field-dressed orbitals rather than the field-free orbitals are considered. Our measured angular distributions and intensity dependence show that these field-dressed orbitals can have strong Rydberg character for certain orientations of the molecule relative to the laser polarization and that they may contribute significantly to the hydrogen elimination dissociative ionization yield. These findings suggest that Rydberg contributions to field-dressed orbitals should be routinely considered when studying polyatomic molecules in intense laser fields.

## Introduction

Strong-field ionization is a key topic in ultrafast science since it is an essential step in attosecond pulse generation^1–5^, serves as a probe of electronic and nuclear dynamics^6–13^ and is used to image molecular orbitals^14–17^. Continued development of our understanding of ionization dynamics in molecular systems^18^ is an important aspect of forefront experimental challenges such as controlling molecular fragmentation dynamics^19–22^, the creation of multi-hole electronic wave packets^23–27^ and the drive for ever finer time-resolved measurements of molecular dynamics^28, 29^ that one day may, for example, probe charge migration^30–33^ on intrinsic timescales.

Strong-field ionization is well studied in atoms^34–41^, in the benchmark H_2_ molecule^42–47^ and in somewhat more complex systems^48–50^. This work has informed our understanding of many characteristic strong-field processes, such as tunnel ionization, above-threshold ionization and high harmonic generation. Since the electronic states in atomic systems are generally well-separated, in many cases the behavior of the outermost occupied orbitals approximately characterize the entire process. As strong-field ionization experiments moved to diatomic molecules, however, the electronic behavior became more complex. Unlike in atomic cases, a simple ionization potential could no longer adequately characterize the relative tunneling rates^48, 51–55^. Subsequent work has included examples of diatomics (CO, N_2_, HCl)^26, 56–58^ where several orbitals participate in the tunneling process. These studies and other recent efforts exploring strong-field molecular ionization of multi-electron systems^56–71^ suggest that a full understanding of the ionization process and associated angular structure requires consideration of not just the highest occupied molecular orbital (HOMO) but also the neighboring HOMO-1. In this study we show that to correctly predict the angular character of strong-field ionization of small hydrocarbon molecules, formerly unoccupied molecular orbitals should be taken into account as well.

Polyatomic molecules are now the focus of many strong-field ionization experiments since these molecules are important in a variety of settings, such as the building blocks in molecular machines, in quantum information applications, for energy storage and structural classification of proteins. These experiments offer opportunities to test imaging techniques^72–75^ and explore and control dynamics^60, 64, 76^ in more complicated molecular systems. The polyatomic nature of the system does not change the foundational role of strong-field ionization in ultrafast processes, but the ionization dynamics become increasingly complex. Mechanisms such as Freeman resonances^77^ and laser-induced AC Stark shifts result in more complicated behavior as the number and proximity of electronic states increase. Of particular interest in this work is the strong-field driven modification of the molecular orbitals, which becomes relatively more important as the number and angular complexity of the molecular orbitals increase and the energetic separation of the field-free orbitals decreases. Field-driven excitation of the orbitals of the constituent atoms in the polyatomic molecule can lead to molecular orbitals that have many characteristics of Rydberg orbitals. Technically, molecular Rydberg states are formed when one of the excited atomic orbitals involved in bonding has a principal quantum number that is higher than the principal quantum number of the conventional atomic orbital. These Rydberg states are usually quite diffuse and centered on the molecule as a whole rather than an individual atom. Common theoretical practice uses only the field-free orbitals to describe ionization, but as recently shown for strong-field ionization of excited cyclohexadiene and its derivatives, the field-dressed orbitals can have altered spatial characteristics^78^ and a significant amount of Rydberg character, which leads to high ionization rates. In this article, we present a series of measurements that illustrate that these Rydberg contributions also play an important role in the strong-field ionization of small hydrocarbon molecules starting from their electronic ground state.

## Results

**Figure 1:**
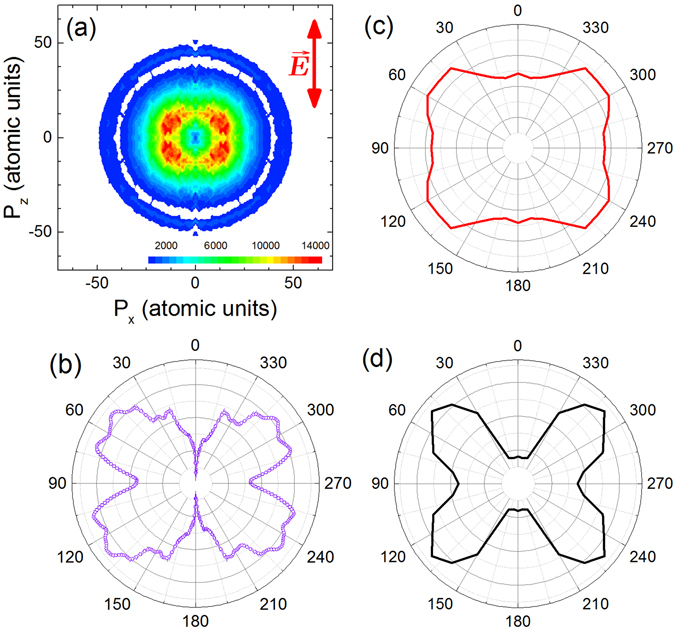
(**a**) The two-dimensional slice (P_y_ ≈ 0) through the three-dimensional momentum distribution obtained from VMI data of C_2_D_3_^+^ photofragments produced in the nω + C_2_D_4_ → C_2_D_4_^+^ → C_2_D_3_^+^  + D process. The laser polarization, indicated by the arrow, is vertical (0–180°) in all panels. The faint outer ring is mirrored in the D^+^ momentum image, suggesting that those ions are part of the double ionization process (D^+^  + C_2_D_3_^+^). The laser pulses are approximately 5 fs in duration with a central wavelength of 740 nm and a focused peak intensity (I_peak_) of approximately 6 × 10^14^ W cm^−2^. The corresponding focal-volume-averaged intensity, I_avg_, (see the Methods section for details) is approximately 2 × 10^13^ W cm^−2^. (**b**) Measured yield as a function of the relative angle between the C_2_D_3_^+^ photofragment and the laser polarization. The yield is obtained for the inner single ionization followed by hydrogen elimination process and excludes the faint outer double ionization process. (**c**) Calculated angular distribution for the C_2_D_3_^+^ photofragments (see Methods for details) without including FDRC orbitals at a uniform intensity of 9 × 10^13^ W cm^−2^. (d) Similar calculations for an intensity of 9 × 10^13^ W cm^−2^ but with the ionization from FDRC orbitals included. The ethylene HOMO has π symmetry.

We focus on a specific process initiated by intense few-cycle laser pulses in acetylene (C_2_D_2_), ethylene (C_2_D_4_), and ethane (C_2_D_6_), namely single ionization of the parent molecule followed by hydrogen elimination. In each case, we measure the momentum of the remaining C_2_D_n−1_^+^ fragment using velocity map imaging (VMI)^79^. We are assured that this fragmentation channel is uniquely identified as neutral hydrogen elimination by the lack of any momentum-matching D^+^ partner ions obtained under the same laser conditions, therefore excluding contributions from the D^+^ + C_2_D_n−1_^+^ channel. The short pulse duration (approximately 5 fs) limits any possibility for significant vibration or rotation of the nuclei while the laser pulse is present^18, 80^ and avoids molecular dynamics such as internal conversion that sometimes occur on excited states of the neutral molecule via multiphoton resonances^81–83^. Thus, the measured C_2_D_n−1_^+^ fragment angular distributions can represent the angle-dependent ionization probability. In the ethylene case, the general four-lobed structure shown in Fig. 1a,b for the C_2_D_3_^+^ photofragments is independent of pulse intensity and duration (at least up to ≈45 fs^76^).

The comparison between measured and calculated C_2_D_3_^+^ photofragment angular distributions from ethylene, shown in Fig. 1, clearly illustrates the need to include Rydberg contributions from field-dressed orbitals if the calculations are to even approximate the experimental result. Calculations (detailed in the Discussion and Methods sections) that do not include ionization from field-dressed orbitals with Rydberg character (called FDRC orbitals from now on) result in an approximately isotropic angular distribution, like the one shown in Fig. 1c. Here the tunnel ionization is considered only from the HOMO. Including ionization from FDRC orbitals yields the four-lobed structure illustrated in Fig. 1d, which qualitatively matches the experimental results. The influence from lower lying orbitals was also examined but unlike CO^60^ or other small molecules^68^, the effect of these orbitals was negligible for the present calculations.

**Figure 2:**
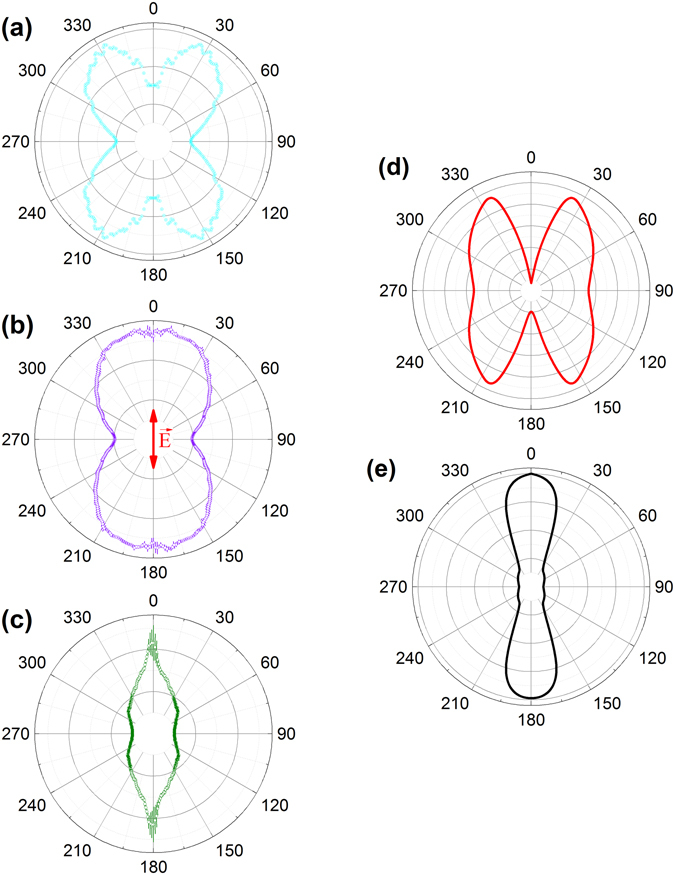
(Left) Experimental C_2_D^+^ photofragment angular distributions arising from nω + C_2_D_2_ → C_2_D_2_^+^ → C_2_D^+^  + D. Experimental laser parameters are about 5 fs pulse duration and a central wavelength of 740 nm. The laser polarization is vertical (along the 0–180° direction) in all panels, as indicated by the arrow. (**a**) Experimental results with I_peak_ = 4 × 10^15^ W cm^−2^ and I_avg_ = 4 × 10^13^ W cm^−2.^ (**b**) I_peak_ = 8 × 10^15^ W cm^−2^ and I_avg_ = 7 × 10^13^ W cm^−2^. (**c**) I_peak_ = 1 × 10^16^ W cm^−2^ and I_avg_ = 1 × 10^14^ W cm^−2^. (Right) Calculated angular distribution for the C_2_D^+^ photofragments (see Methods for details). In panel (d) the calculations are done without including FDRC orbitals, at a uniform intensity of 9 × 10^13^ W cm^−2^. (**e**) Similar calculations but with the ionization from FDRC orbitals included, at the same intensity of 9 × 10^13^ W cm^−2^. The symmetry of the acetylene HOMO and LUMO are π_u_ and π_g_, respectively.

**Figure 3:**
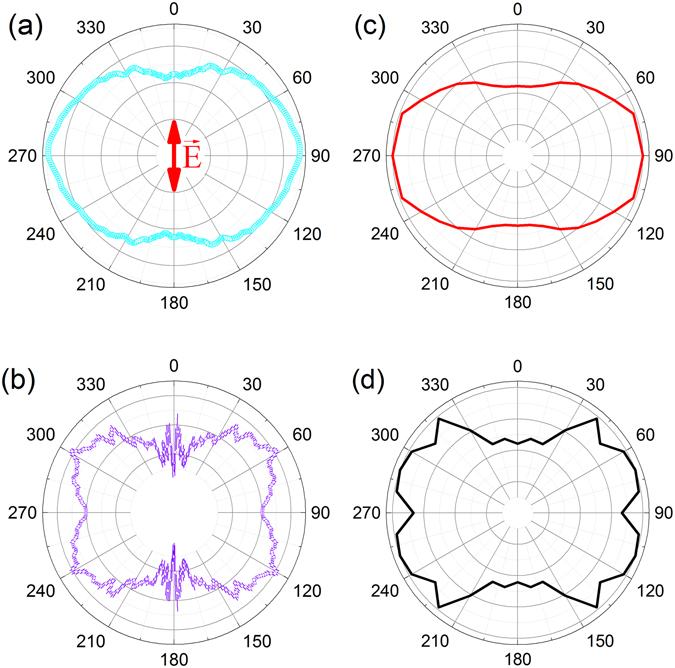
(**a**) Measured C_2_D_5_^+^ photofragment angular distribution from the nω + C_2_D_6_ → C_2_D_6_^+^ → C_2_D_5_^+^ + D process at I_peak_ = 2 × 10^15^ W cm^−2^ (I_avg_ = 2 × 10^13^ W cm^−2^) and a pulse duration of about 5 fs. (**b**) Measured C_2_D_5_^+^ photofragment angular distribution for the same process and pulse duration but at a higher intensity: I_peak_ = 7 × 10^15^ W cm^−2^ and I_avg_ = 6 × 10^13^ W cm^−2^. (**c**) Calculated C_2_D_5_^+^ angular distribution without including FDRC orbitals, at a uniform intensity of 9 × 10^13^ W cm^−2^. (**d**) Calculated C_2_D_5_^+^ angular distribution once the FDRC orbitals are included, at a uniform intensity of 2 × 10^15^ W cm^−2^. The laser polarization, indicated by the red arrow in (**a**), is vertical in all panels. The ethane HOMO has π* symmetry.

Similar calculations to those performed for ethylene were conducted for the hydrogen elimination channels in acetylene and ethane, nω + C_2_D_2_ → C_2_D_2_^+^ → C_2_D^+^ + D and nω + C_2_D_6_ → C_2_D_6_^+^ → C_2_D_5_^+^ + D, respectively. In those two cases we can observe the intensity dependent “turn on” of contributions to the ionization from the FDRC orbitals. The comparison between the calculations and the experimental results are shown in Figs 2 and 3. For acetylene the calculations for the angular distribution are depicted in Fig. 2d,e, where 2(d) only takes the HOMO into account, while 2(e) also includes the FDRC orbital. In theory a clear cutoff intensity exists, above which the FDRC orbital becomes partially occupied in the field and therefore the angular distribution takes the form of Fig. 2e, while below the cutoff intensity only the HOMO is occupied and the distribution is the shape of Fig. 2d. These idealized conditions cannot be replicated in the experiment as the intensity of the laser varies over the focal volume. Therefore in the experiment both cases of molecules, those who only ionize from the HOMO and those who have the FDRC orbital partially occupied, contribute to the measured data. With increasing intensity the number of molecules exhibiting a FDRC contribution rises so the shape gradually goes from 2(d) to 2(e). Figure 2a–c shows exactly this, with increasing intensity the angular distributions come to resemble 2(e) more and more. Experimentally we note the larger error bars along the polarization axis in Fig. 2c. This noise, which is discussed in the methods section, does not affect the general conclusion that the photofragment angular distribution narrows at higher laser intensity.

The experimental and theoretical results for ethane molecules are shown in Fig. 3. Here the effect of “turning on” the contributions to the ionization from the FDRC orbitals can be seen as well in the four lobes observed at higher intensity (Fig. 3b). Since the shapes with and without FDRC contributions, shown in Fig. 3c,d, are more similar than in the acetylene case the gradually shifting effect on the angular distributions is not as easily visualized. The intensity dependence is described further in the Discussion section. Clearly, the angle-resolved ionization from all three of these small hydrocarbon molecules show significant effects due to contributions from the FDRC orbitals that are populated in intense, few-cycle laser pulses.

## Discussion

The angle-dependent ionization probabilities for ethylene, acetylene and ethane shown in Figs 1, 2 and 3 are calculated based upon electronic structure theory including the laser field as an external dipole field in the Hamiltonian, as detailed in ref. 68 The neutral molecules in our effusive jet are randomly oriented, and thus the interaction between the laser field and the electronic wavefunction depends on their angle relative to the laser polarization. In the calculations, therefore, the molecule is rotated in the plane formed by laser polarization and the C = C bond, as well as out of the plane, i.e., around the C = C bond axis. For each position, the electronic wavefunction is calculated in the presence of the electric field and the tunneling probability is deduced for various orbitals. Coherent ionization from multiple orbitals is treated using a linear combination of the selected orbitals, as described in ref. 68

**Figure 4:**
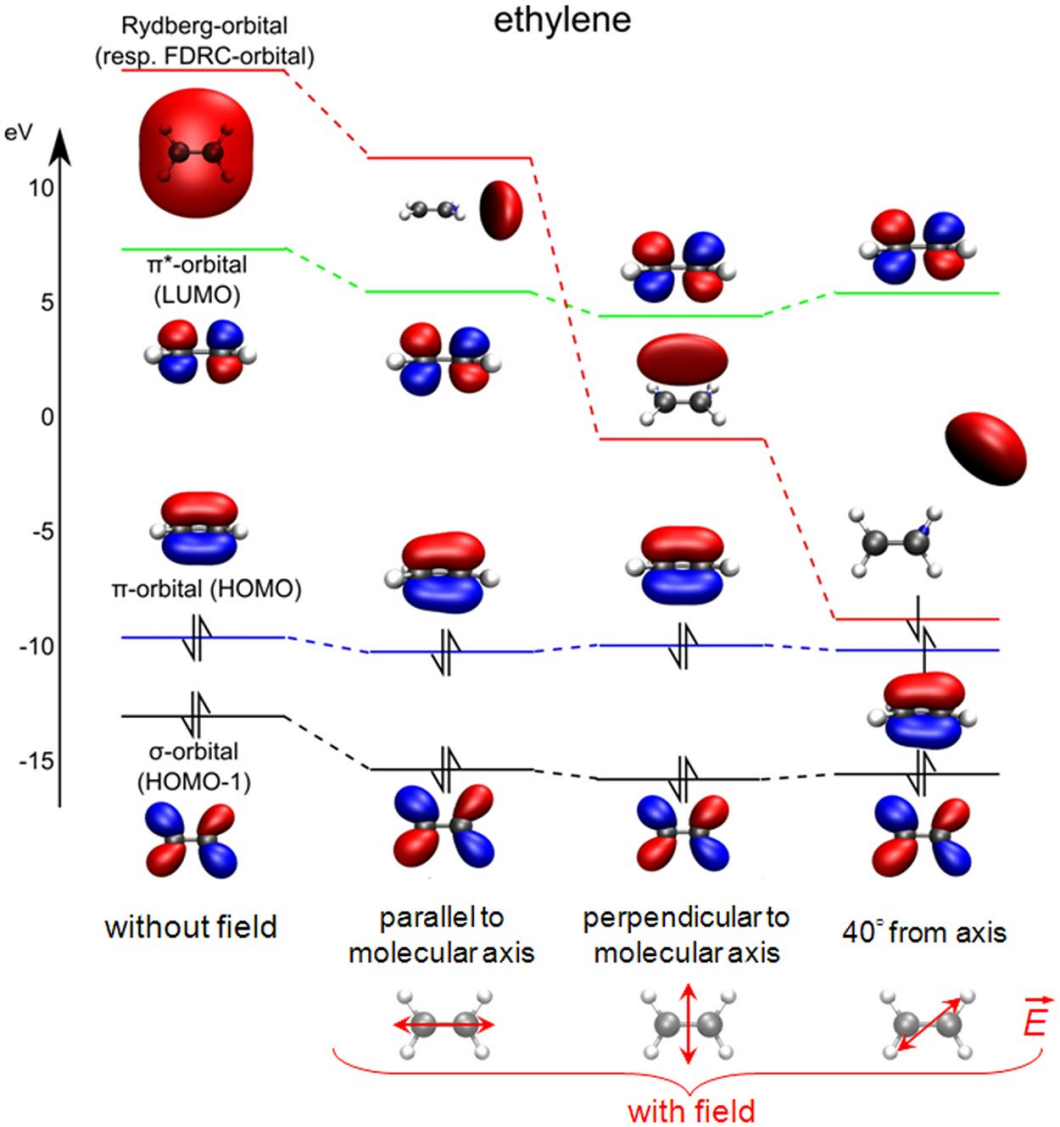
Ethylene orbitals for various orientations of the laser polarization, for an intensity of 9 × 10^13^ W cm^−2^. The rightmost column shows the case where the laser polarization lies along the C-H bond direction. In this configuration, the field easily shifts electron density in that direction and the ionization rate correspondingly increases.

As illustrated in Fig. 4, in the case of ethylene, the σ-, the π- and the π* orbitals do not change significantly either in shape or in energy under the influence of the laser field. In contrast, the orbital with Rydberg character reacts strongly to the applied field. Its shape aligns with the direction of the laser field, and its orbital energy strongly depends on the laser polarization. For polarization parallel or perpendicular to the C = C backbone, the orbital energy is lowered, but the HOMO and the FDRC orbital remain well separated in energy. When the laser polarization is aligned with the C-H bond direction, however, the energy gap between these orbitals decreases significantly. These calculations indicate that the field stabilizes a high-lying Rydberg orbital with a localized electron density in the direction of the laser polarization. For these orientations of the molecule relative to the laser polarization, the stabilization of the Rydberg orbital is so large that it becomes energetically close to the field-dressed π orbital (HOMO), leading to partial occupation of the FDRC orbital in the laser field. The electron density is far from the nuclei and the tunneling ionization rate becomes relatively large.

The calculated angular-dependent tunnel ionization only describes the electron leaving the system, while the experimental measurement is the photofragment from a C-H dissociation. To compare the theoretical calculations with the experimental results, we need to calculate the C-H bond dissociation direction related to the angular-dependent ionization rate. For the smallest molecule C_2_H_2_, the correlation between the detected C_2_H^+^ fragment and the angular-dependent ionization rate is simple, as this molecule is linear. If for example the molecule is rotated by an angle α with respect to the laser polarization and its ionization leads to a C-H bond break, the detected signal will be at the angle α (or 180° + α). The rotation of the molecule is about 1000 times slower than the vibration correlated with the dissociation and does not significantly influence the shape of the angular distribution of the fragments. Thus the angular distribution is the same for the electron leaving the system as for the neutral hydrogen or the C_2_D^+^ fragment leaving the system.

For the other two non-linear molecules with more than two H-atoms attached, the case is more complex. Therefore, we introduce a mapping of the tunnel ionization to the photofragment dissociation direction. In our calculations we use the C-C axis to determine the position of the molecule relative to the laser.

**Figure 5:**
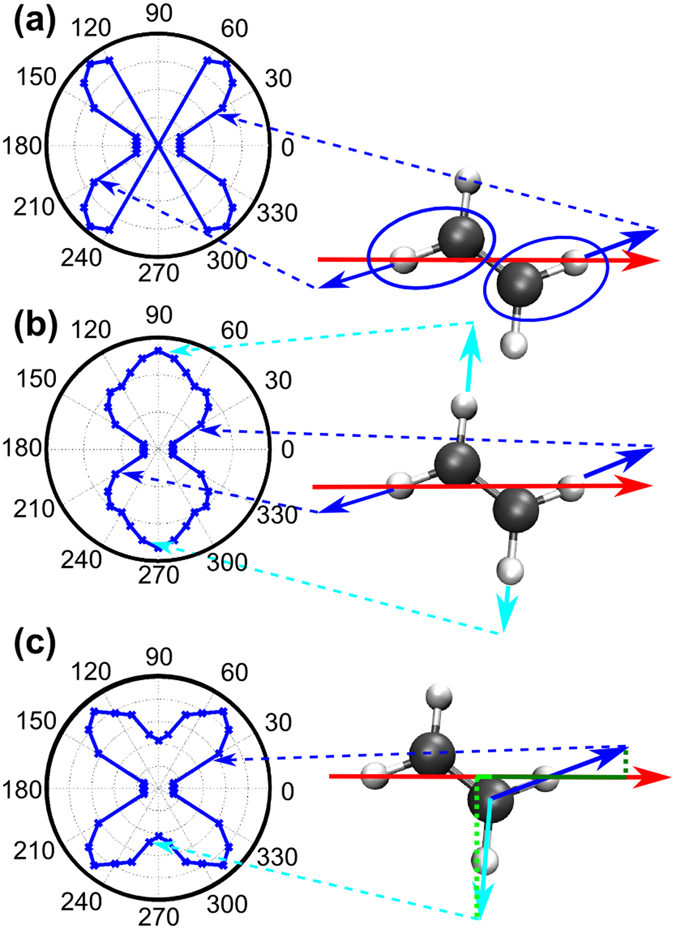
Schemes for calculating the photofragment angular distributions in C_2_D_4_ (right) and corresponding results (polar plots, left). The red arrow indicates the direction of the dipole field. The dashed arrows relate the orientation of the molecule to the dipole vector with the angle between the field and the dissociation direction plotted in the polar diagram. (**a**) Contributions from the hole localized at “nearby” C-H bonds only. The blue vectors indicate the dissociation direction of these H-atoms. (**b**) The hole delocalizes quickly, allowing all C-H bonds to dissociate with equal probability. The cyan vectors indicate the dissociation direction of the more remote H-atoms (**c**) Contributions to dissociative ionization yield are weighted partially by their projection to the dipole vector which is indicated by the dark and light green line, respectively (see Eq. (4)). The individual projections are indicated as dotted lines.

In calculating the angular distributions for hydrogen elimination in C_2_H_4_, we have explored three possible dissociative ionization scenarios, which are illustrated in Fig. 5a–c. In all scenarios tunnel ionization creates an electronic wavepacket with a hole localized along the two C-H bonds that align most closely with the laser field (“nearby” C-H bonds). The three scenarios differ in the degree of influence the localized wavepacket has for preferential C-H bond breaking.

In the first scenario, the tunnel ionization probability is largest when a C-H bond is aligned along the laser polarization. This situation leads to subsequent dissociation of these nearby C-H bonds. In this scenario, the hole is not allowed to evolve from its birthplace along the direction of the laser field and it is assumed that the nearby bonds break and not the other C-H bonds. In this scenario the localized wavepacket has a great influence in selecting possible C-H bond breaks. As can be seen in Fig. 5a, the theoretical photofragment distribution for this scenario displays a four-lobed structure similar to that observed in the experimental data. This scenario, however, as expected, yields zero ionization for 60°−120° and 240°−300°, as these angles do not correspond to the C-H bonds being near the laser polarization.

In the second scenario the electronic wavepacket delocalizes almost instantaneously and all C-H bonds dissociate with the same probability. In this scenario the former localized wavepacket has no influence on selective C-H bond breaking. Figure 5b shows the predicted angular distribution for this scenario. The yields at 90° and 270° come from dissociation of the C-H bonds farthest from the polarization axis of the laser field, while the smaller contributions at angles of 45°, 135°, 225° and 315° are due to dissociation of the nearby C-H

**Figure 6:**
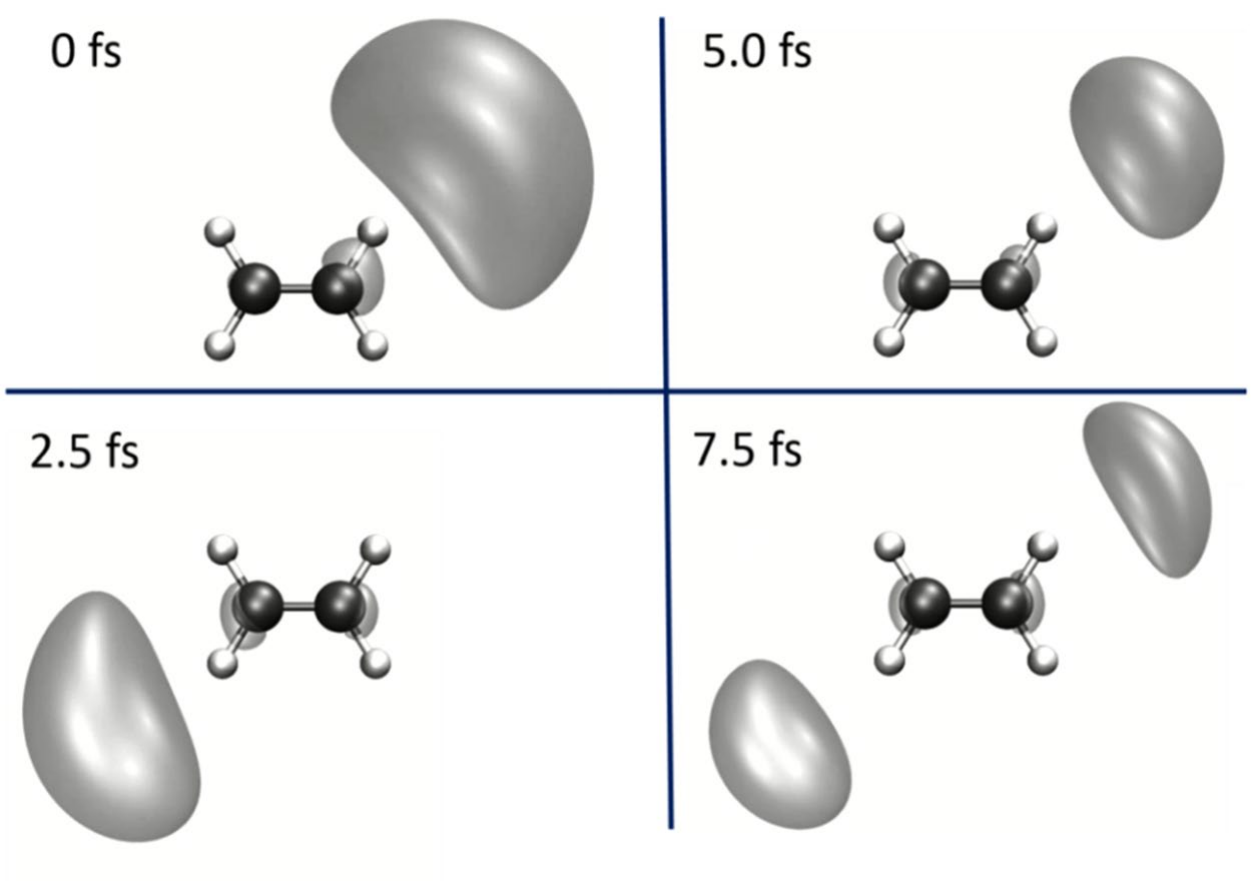
Snapshots of the time-dependent hole density in ethylene. The complete animation produced in the calculation is included in the supplemental information.

As in the aforementioned cases, the final scenario, illustrated in Fig. 5c, dictates that the probability of hole creation is favored for the nearby C-H bonds, but here the photofragment yield contributions from various sites are weighted by their “distance” to the laser field. This distance is indicated by the dotted green lines in Fig. 5c. In this sense this scenario is a middle ground between scenario one and two. The localized electronic wavepacket influences the selection of which C-H bond breaks, in contrast to scenario two, but not as exclusively as in scenario one. Hence, while the nearby C-H bonds are most likely to break, the other C-H bonds have a much smaller but non-zero probability of breaking as well, thus leading to the yields at 60°−120° and 240°−300°, seen in Fig. 5c, which were absent in scenario 1. The final scenario best matches the experimental result, and its underlying idea is supported by the time-dependent propagation of the hole in the electronic density, shown in Fig. 6. The electron hole density (after ionization) is demonstrated to spend the most time in the vicinity of the nearby C-H bonds within the first vibrational period of about 10 fs (the vibration is not shown). An animation of the electron hole density for the first 10 fs after ionization is available as supplemental information to this article. A similar strategy was applied to the ethane case in order to obtain the photofragment emission angular-distributions.

**Figure 7:**
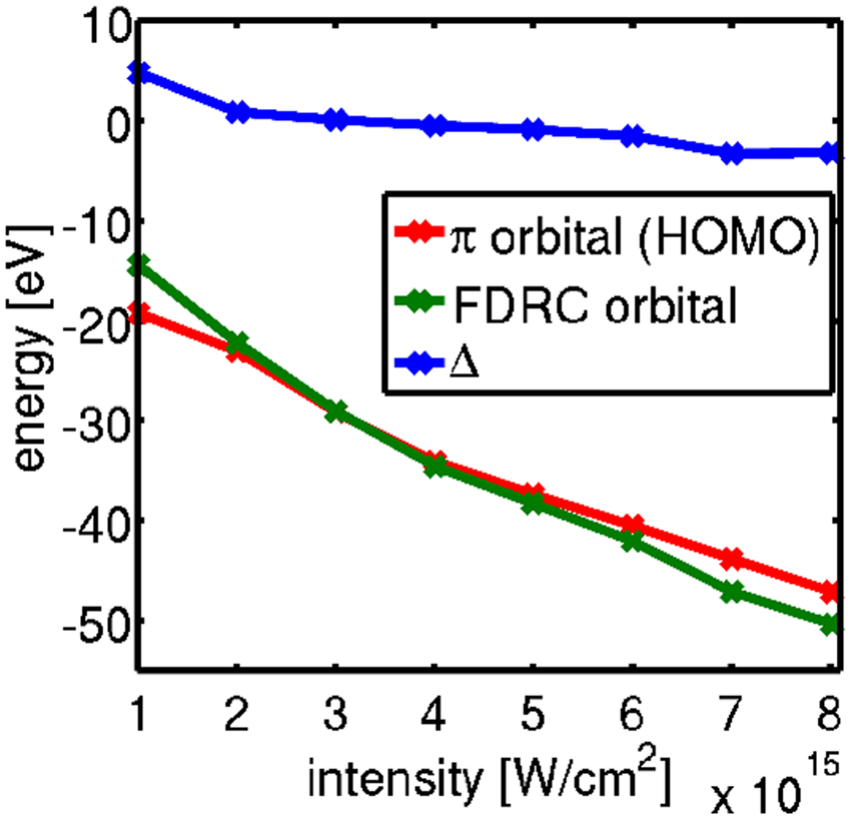
Intensity-dependent energies of the HOMO (red line) and the FDRC orbitals (green line) in ethane. The laser polarization is along a C-H bond for the most noticeable effect. Δ represents the difference in energy between these orbitals as a function of intensity and is shown by the blue line.

As shown in Figs 2 and 3, the FDRC orbitals make an important contribution to the dissociative ionization in acetylene and ethane. In those cases the effect of “turning on” the contribution of the FDRC orbital can be clearly seen. In the ethylene case the HOMO-only angular photofragment distribution, shown in Fig. 1c, is nearly isotropic and therefore does not make an easily observable contribution to the measured angular distribution. This effect masks the intensity-dependent transition from the HOMO-only photofragment angular distribution to the angular distribution where the FDRC orbitals become relevant. The energy gap separating the HOMO and the FDRC orbital defines the intensity at which the FDRC orbitals are partially occupied and contribute significantly to the ionization yield. This idea is supported by Fig. 7, which shows the intensity-dependent energies of the field-dressed HOMO and the FDRC orbitals of ethane. With increasing intensity the orbitals come close in energy and eventually cross. The intensity at which the FDRC orbitals in ethane should become relevant is at around 3 × 10^15^ Wcm^−2^. Depending on the molecules in the volume of the laser focus above the crossover intensity the observed distribution shifts from the predicted shape with HOMO only to the calculated form that includes the FDRC orbitals.

A final point that this study can begin to probe is the usefulness of the molecular orbital picture for examinations of strong-field ionization for these examples as well as other large molecules^84^. Despite the fact that the MO-ADK^73^ and other ionization models (e.g. ref. 68) based on molecular orbitals have been fairly successful, describing the ionization in terms of field-free molecular orbitals is strictly valid only when Koopmans’ theorem holds; *i*.*e*. within Hartree-Fock theory, the first ionization energy is equal to the negative of the HOMO orbital energy. This condition is not met in the case of many polyatomic molecules. In these cases, the preferred methods for treating the ionization of a single electron include the use of a Dyson orbital^85^ or including “dynamic exchange” effects^86^ in the calculation of the time-dependent Schrödinger equation. Calculation of the Dyson orbital requires evaluating the overlap between the multiple-electron wavefunctions of the neutral and the cation. Dynamic exchange calculations of strong-field ionization of molecules include effects beyond the relative symmetry of the initial and final states; specifically these calculations take into account the antisymmeterization of the virtual states that occur during the ionization process^86^. Both of these techniques go beyond a field-free description of the electronic characteristics of the target molecule. In the calculations presented here, we also go beyond the field‐free molecular orbital approach by calculating the molecular orbitals with and without field at the CAS level of theory to capture the important part of the electronic wavefunction and allow ionization to occur from more than one orbital. The field-dressed molecular orbitals describe much of the electron correlation that occurs when the bound electronic states are coupled by the strong laser field to the ionized continuum states. The model, however, does not go so far as to make a full Dyson orbital calculation or include dynamic exchange effects.

Despite the various potential theoretical liabilities discussed above, does the current model produce acceptable results? If the problem depended on the behavior of the electron during the tunneling process it is likely that the model would be insufficient because the molecular orbitals would not be precise enough. Importantly, however, the present problem only depends on the angular-dependent tunneling probabilities, and these are quite accurate. By using field-dressed molecular orbitals rather than simply mixing field-free orbitals, electron correlation effects are included in the total electronic wavefunction that is subsequently used to depict the molecular orbitals relevant to the tunnel ionization. The agreement between the calculations and the experiment despite the various approximations and assumptions suggests that the somewhat simplified model presented here reproduces most, if not all, of the important aspects of the strong-field ionization. A more careful comparison of these different theoretical methods is a potential pathway for future work. It should be noted that the involvement of the FDRC orbitals is a property of the (studied) hydrocarbons and is not present in simpler diatomic molecules like CO^60^, although the necessary basis functions to form FDRC orbitals were available in previous calculations^68^.

Collectively, these results provide robust evidence of the important role played by field-dressed orbitals with Rydberg character in strong-field ionization of molecules where the energy separation between the HOMO and the FDRC orbitals is comparable to the Stark shift caused by the laser. These conditions are satisfied in the hydrocarbon molecules studied here and should be relevant for large classes of polyatomic molecules that are attracting increasing experimental interest. Ionization from these field-dressed orbitals with Rydberg character creates holes in the electronic wavefunction that strongly influence the direction of the hydrogen elimination from the molecular cation. Understanding the link between the electronic properties of the ionization process and the photofragment angular distributions that result from molecular dissociation is an essential component of designing adaptive control schemes that use photofragment imaging as a feedback source^76^.

## Methods

### Calculations

We performed quantum chemical calculations for the ground state with the MOLPRO program package^87^ at the CASSCF [10, 12], CASSCF [12, 12] and CASSCF [14, 12] level of theory, for acetylene, ethylene and ethane respectively using the 6–31++ G** basis set. The calculations were carried out with and without an external dipole field. The dipole field was added to the one-electron Hamiltonian to simulate the interaction with the strong ionization field, which corresponds to a static field. While this basis set is at the lower limit of what is suitable for calculations at these field strengths, it is sufficient to demonstrate the influence of the FDRC orbitals.

The ionization probability of a molecule in a laser field can be modeled in terms of the induced electron flux through the barrier of the combined molecular and external electric field^83^ (atomic units *m* = *ħ* = *e* = 1 are used throughout the paper):

1 $$\begin{array}{c}W(t)={\int }_{S}j(r,t)dS,\\ j(r,t)=-\frac{i}{2}({\psi }(r,t)\nabla {\psi }{(r,t)}^{\ast }-{\psi }{(r,t)}^{\ast }\nabla {\psi }(r,t))\end{array}$$

Here *j(r*,*t)* is the electron flux density and *ψ(r*,*t)* is the electronic wavefunction in the presence of the electric field inducing the electron flux *W(t)*. For the surface *S* it is convenient to choose a plane perpendicular to the direction of the electric field. In our case, *S* is located at the outer turning points of the electronic wavefunction. Here the wavefunction enters the classically-forbidden region where tunneling occurs. The electronic wavefunctions, evaluated by a quantum chemical program package, are typically real, and their flux density (Eq. 1) is zero.

Refs 68 and 88 demonstrated that this problem can be overcome by evaluating the electron flux for the electron density *ρ(r*,*t)* with the help of the divergence theorem and the continuity equation, as proposed by^88^.

We can then rewrite Eq. (1) as:

2 $$W(t)=-{\int }_{V^{\prime} }\nabla j(r,t)dV^{\prime} =\frac{d}{dt}{\int }_{V^{\prime} }\rho (r,t)dV^{\prime} $$

with *V′* being the part of the total volume *V* in which the electronic wavefunction *ψ(r)* is defined and which is spanned by the surface S and a vector perpendicular to S pointing away from the nuclei. In order to calculate the tunneling probability *T(S)*, we need the electron density with (at final time *t*_*f*_) and without the external field (at initial time *t*_*i*_).

Therefore we integrate Eq. 2 over time and obtain the following:

3 $$T(t;S)={\int }_{V^{\prime} }\rho (r,{t}_{f})dV^{\prime} -{\int }_{V^{\prime} }\rho (r,{t}_{i})dV^{\prime} $$

To treat ionization from more than one single orbital we solve the working equations derived above for a linear combination of the selected molecular orbitals. This implies a basis transformation rewriting the two orbitals (e.g. HOMO and LUMO) in the Slater determinant as the orbitals HOMO + LUMO and HOMO - LUMO, allowing for coherent ionization of the electron from both orbitals^60, 89^. The coefficients for the linear combination are taken from the CASSCF calculations and correspond to the coefficients of the configuration expansion of the ground state electronic wave function.

The position of the molecules relative to the dipole field is given by a rotation φ along the C-C axis and a rotation *α* perpendicular to the C-C axis. Both rotations were varied in 10° steps. The resulting tunnel ionization *T*(*α*) is obtained from Eq. 3 by integrating over the angle φ, as the surface S can be written depending on the angles *α* and φ.

**Figure 8:**
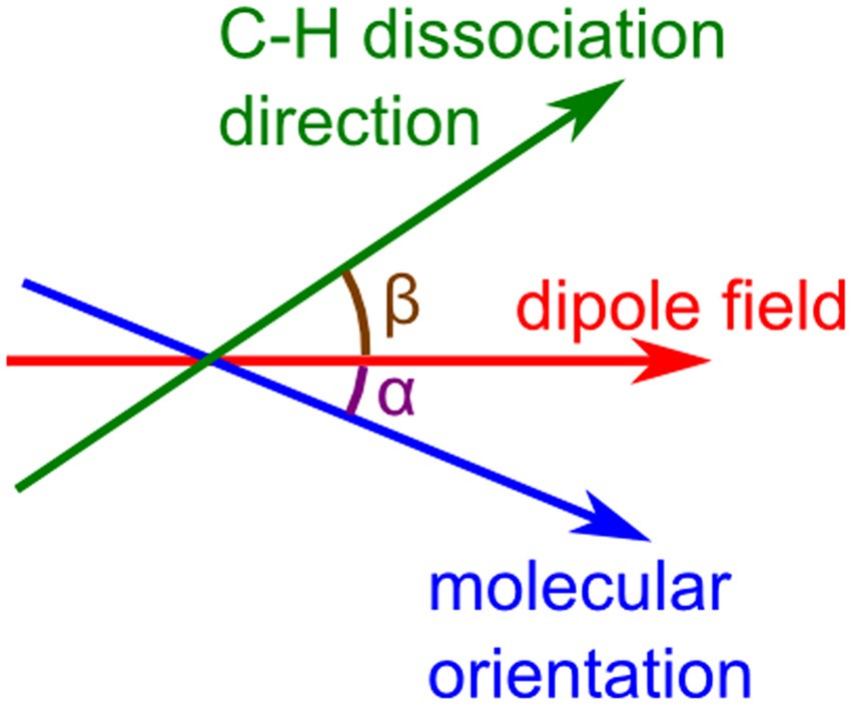
Relevant angles α, between the dipole field and the molecular orientation (in this case the C-C axis), and β, between the dipole field and the C-H dissociation direction.

Once the angular dependent tunnel ionization is obtained, a mapping between that quantity and the much slower C-H (C-D) dissociation is needed for the non-linear C_2_D_4_ and C_2_D_6_ molecules. For the mapping step from angular tunnel ionization *T*(*α*) to the calculated angular H^+^ fragment signal *U*(*β*), where β describes the angle between the dipole field and the C-H dissociation direction, as shown in Fig. 8, we used the following formula:

4 $$U(\beta )=\sum _{\alpha }T(\alpha )\cdot M(\alpha ,\beta )\cdot w(\alpha ,\beta ),$$

with *M*(*α*,*β*) the mapping function and *w*(*α*,*β*) a weight for the “distance” between the C-H bond and the dipole field. The mapping function is one if β coincides with a C-H bond for a given angle α and zero otherwise. The weight was set to $w(\alpha ,\beta )=0.5(1+\frac{\langle \overrightarrow{V}|\overrightarrow{D}\rangle }{|\overrightarrow{V}||\overrightarrow{D}|})$, where $\overrightarrow{V}$ is the vector along the dissociation direction shown as the blue (and cyan) vectors in Fig. 5c, and $\overrightarrow{D}$ is the vector of the dipole field.

The tunnel ionization creates a hole in the electron density in the FDRC orbital. This orbital is taken to represent the hole. To visualize the hole dynamics, the FDRC orbital is projected onto the basis of the field free orbitals. This projected hole is treated like an electronic wavepacket and propagated in the eigenstate basis.

### Experiment

A Ti:Sapphire laser system (named PULSAR) generates 2 mJ pulses of approximately 24 fs duration and 790 nm central wavelength at 10 kHz. These pulses are used to generate few-cycle pulses (~200 μJ, 450–1000 nm bandwidth) through self-phase modulation in an argon-filled hollow-core fiber. A set of chirped mirrors compensates for substantial up-chirp acquired during spectral broadening and creates an overall negative dispersion to counterbalance positive dispersion from propagation through air and glass in the beam path. Moreover, a pair of fused silica wedges allows for fine adjustment of the dispersion. These procedures allow delivery of Fourier transform-limited pulses of about 5 fs duration on target. A small fraction of the laser beam is split off and focused into a stereographic above-threshold ionization (ATI) phase meter^90, 91^. Using the phase meter to obtain a parametric asymmetry plot of the expected radius verifies the production of few-cycle pulses^92^. The main portion of the laser beam travels to the velocity map imaging (VMI)^93, 94^ spectrometer and is focused by an *f* = 75 mm spherical mirror inside the chamber. An iris placed just before the entrance is used to vary the peak laser intensity, which is evaluated approximately using simple Gaussian beam optics.

Our VMI spectrometer follows well-documented design and operation, e.g.,^79, 95^. An effusive gas jet of the target hydrocarbon molecules intersects the laser beam inside the spectrometer, which is composed of an electrostatic lens system that focuses photofragments to specific radii on the detector depending upon their transverse momenta. Using a fast high voltage switch, the detector is active during a narrow time window (80–100 ns wide) around the expected arrival time of the fragment of interest. Deuterated hydrocarbon gas is used to ensure adequate time separation of fragments differing by only one “hydrogen” atom. The obtained images are inverted offline using a version of the onion-peeling method^79, 96^ to retrieve the two-dimensional slice (P_y_ ≈ 0) through the three-dimensional momentum distribution of the dissociating fragments.

At the lowest intensity (Fig. 2a) measurement for the acetylene target, the nω + C_2_D_2_ → C_2_D_2_^+^ → C_2_D^+^ + D^+^ double ionization channel is negligible. At higher intensities this channel is present, but well separated (similar to the ethylene case in Fig. 1a) from the C_2_D^+^ + D single ionization channel of interest and does not significantly affect the angular distribution shown in Fig. 2b. At the highest intensity (Fig. 2c), however, the double ionization channel dominates the outside of the image and thus somewhat compromises the VMI inversion process. The 12-bit dynamic range of the camera that images the phosphor screen in our setup limits the image acquisition time so as not to saturate the portion of the detector collecting the larger-momentum higher-yield C_2_D^+^ photofragments arising from double ionization. The corresponding signal from lower-momentum, smaller-yield C_2_D^+^ photofragments arising from single ionization is small and also contains contributions from higher momentum C_2_D^+^ photofragments arising from double ionization that must be subtracted in the image inversion process^79, 96^. The small signal along with numerical uncertainty from the inversion leads to noisy data along the polarization axis, as indicated by the error bars in Fig. 2c. Despite this problem along the center of the image, the data that is more than a few degrees away from the polarization direction demonstrates the change in the photofragment angular distribution discussed in the results.

To compare laser intensity between the calculations and the measurements, we use the measured input beam parameters and the focusing conditions to calculate an experimental volume-averaged intensity. The spread of the effusive jet from the 100 μm opening is much larger than the size of the laser focus at the interaction point. Using Gaussian beam optics, the measured beam waist radius outside the vacuum chamber (${W^{\prime} }_{0}$) and the central wavelength of the laser pulse (*λ*), the beam waist at the focus of the spherical mirror (*W*_0_) is calculated using ${W}_{0}=\frac{\lambda f}{\pi {W^{\prime} }_{0}}$ since the depth of focus of the input beam may be considered to be much longer than the focal length of the mirror (*f*)^97^. The optical intensity is then a function of the radial distance *z* and the axial distance $\rho ={({x}^{2}+{y}^{2})}^{\frac{1}{2}}$,

5 $$I(\rho ,z)={I}_{0}{[\frac{{W}_{0}}{W(z)}]}^{2}\exp [-\frac{2{\rho }^{2}}{{W}^{2}(z)}]$$

where *I*_0_ is the peak intensity,

6 $$W(z)={W}_{0}{[1+{(\frac{z}{{z}_{0}})}^{2}]}^{\frac{1}{2}}$$

and *z*_*0*_ is the Rayleigh range

7 $${z}_{0}=\frac{\pi {W}_{0}^{2}}{\lambda }=\frac{\lambda {f}^{2}}{\pi {({W^{\prime} }_{0})}^{2}}.$$

The focal volume is then defined as the region where *I*(*ρ*,*r*) is larger than an experimentally determined value of the peak intensity that produced little or no signal of the photofragment of interest. *I*(*ρ*,*r*) is then averaged over this volume to obtain I_avg_, the focal-volume-averaged experimental intensity. This value is used along with the peak intensity (I_peak_) to compare to the intensity used in the calculation.

## Electronic supplementary material

Supplemental Video
